# Global initiatives to improve clinical investigation efficiency: a scoping review of implemented system-level reforms

**DOI:** 10.3389/fmed.2026.1913657

**Published:** 2026-08-17

**Authors:** Ali McDonnell, Majella Geraghty, Olivia McDermott, Tom Melvin

**Affiliations:** 1 School of Medicine, University of Galway, Galway, Ireland; 2 School of Medicine, Trinity College Dublin, Dublin, Ireland; 3 Institute for Clinical Trials, University of Galway, Ireland

**Keywords:** clinical investigation efficiency, clinical trial initiation, process standardisation, regulatory innovation, system-level reform

## Abstract

**Background:**

The process for initiating a clinical trial is lengthy, expensive, and often involves a large amount of redundant work. There has been a large number of programs, reforms and reviews carried out by regulators, academics, government groups and other stakeholders globally to identify the barriers to and make recommendations for enhancing initiation of clinical trials and improving their efficiency with comparatively little success. This study aimed to identify key initiatives implemented to date and determine the critical factors contributing to their success.

**Methods:**

This study utilises a scoping review of a wide range of data sources to ascertain what are the key programs undertaken to date to improve clinical trial initiation and to understand the critical success factors of these. Following screening and selection, 27 data sources from 19 relevant programs were included in this review. Sources were included if they were an initiative that had been implemented, in any jurisdiction, to improve the efficiency of the trial initiation process, for medical devices or pharmaceuticals. Included sources were analysed to identify common characteristics, approaches, and determinants of successful implementation.

**Results:**

Most initiatives aimed at improving clinical trial efficiency were identified in the United States, despite the issue being global in scope. The majority of programs are regulatory bodies or research groups or a combination of both. Key determinants of success included the stakeholders involved, as well as the resources and approach to increasing efficiency. Programs reporting success attributed this to enthusiastic stakeholders from diverse backgrounds and who had the authority to implement the planned changes. Effective resource utilisation included pooling expertise and infrastructure from large networks, adopting new technologies, and employing dedicated staff. A key aspect of the approach was to create a standardised and centralised system.

**Conclusion:**

Efforts to improve clinical trial initiation have predominantly been concentrated in the United States and are commonly driven by collaborative regulatory and research-focused initiatives. Successful programs are characterised by multidisciplinary stakeholder engagement, operational authority, shared resources, technological integration, and centralised standardised systems. These findings may inform the development of future strategies to improve the efficiency of clinical trial initiation globally.

## Graphical abstract

**Figure:**
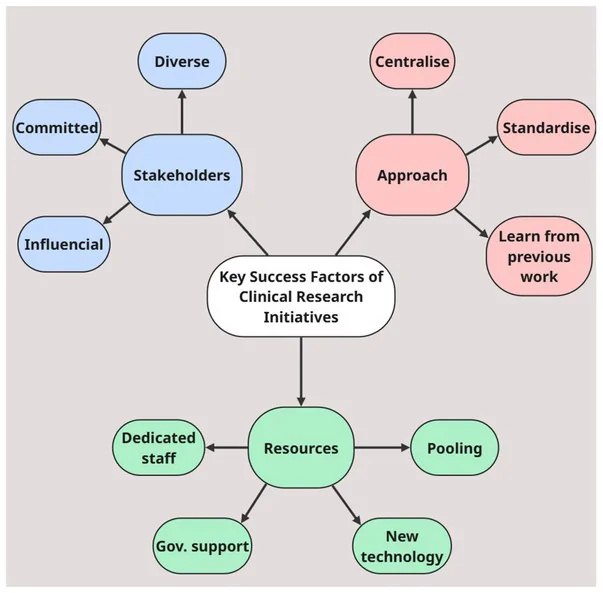
Key success factors for improvements to clinical trial system efficiency.

## 1 Background

Clinical trials are an essential part of the development of pharmaceuticals, medical devices, and surgical interventions, providing critical data needed to evaluate their safety and efficacy. Multiple stages of trials are often needed to bring a new medication or device to market and are beneficial for patients, both those in the trial and those that will be treated with the new intervention later.

The process of gaining approval for and conducting a clinical trial changes depending on the intervention type, purpose, and jurisdiction. However, what is consistent across all clinical trials is the potential for a lengthy process, regulatory confusion, low recruitment rates, and high costs (1). Clinical trials are widely recognised as resource-intensive and operationally complex, with inefficiencies arising from recruitment challenges, protocol complexity, and lack of standardised performance metrics. These issues contribute to delays, increased costs, and reduced trial success rates, highlighting the need for system-level reforms (2).

The developments in regulations surrounding clinical trials and clinical evidence necessary for market approval have increased in recent years. There is also a dramatic increase in costs, which many smaller companies cannot afford, or patients cannot afford to wait years for market access. In 2013, the average time for a medical device to get to market was 3–7 years, compared to the much higher 12 years for pharmaceuticals (3). This has led to various academic, regulatory and patient groups, industry organisations, and government bodies trying to improve the efficiency of trial set up and increase the number of trials being conducted. The ways in which the efficiency was improved varied, but all agree that there is unnecessary work and delays that need to be removed to improve healthcare. Some of the approaches included increased engagement between sponsors and regulators, streamlining processes, and use of new technology.

Previous evidence syntheses have primarily examined barriers to clinical trial delivery (1, 2), including recruitment challenges, regulatory requirements, ethics approval processes, and operational inefficiencies. Other reviews have evaluated specific interventions, such as digital technologies, decentralised trial approaches, or recruitment strategies (3), often within particular therapeutic areas or trial phases. While these reviews have advanced understanding of individual components of trial efficiency, they provide limited insight into broader system-level initiatives that combine multiple interventions or involve coordinated changes across stakeholders. Consequently, there remains a lack of comprehensive evidence describing what initiatives have been implemented internationally to improve clinical trial initiation and what characteristics are associated with successful implementation, rather than current existing reviews which focus on identifying barriers. To our knowledge, there are no previous reviews synthesising implemented initiatives to improve clinical trial initiation efficiency. Unlike prior work, this review focuses on applied reforms rather than theoretical barriers.

Given the breadth and heterogeneity of this evolving landscape and system-level interventions, there is a need to systematically map the initiatives that have been conducted and to learn from them, identifying common success factors, and a scoping review was deemed the most appropriate method (4). Unlike systematic reviews, which focus on narrow questions, a scoping review is well suited to exploring broad topics, identify key concepts, and examine a range of available evidence (5). This review aims to give a comprehensive overview of the initiatives that have been implemented globally to improve clinical trial efficiency, and to determine if there are common success factors. The word ‘initiative’ is used in this review to encompass projects, groups, and programs which implemented changes to the initiation process. The primary research objective was to determine what initiatives have been implemented to improve clinical trial initiation system efficiency or increase clinical trial rates. The secondary research objective was to identify the key success factors for an initiative to increase trial initiation efficiency.

## 2 Methods

The Joanna Briggs Institute (JBI) guidance for scoping reviews was followed, known as the Peters et al. framework (6), which builds on the original Arksey and O’Malley framework for scoping reviews (5). Both adopt a rigorous process of transparency, which enables replication of the search and increases the reliability of findings. The iterations of the frameworks by Peters et al. have added more detail to the stages, guidance on when a scoping review is appropriate, and incorporated the PRISMA-ScR reporting guidelines with a checklist of items to include as part of a scoping review, used in this review and shown in the Additional file 1 (7).

The five stages of Arksey and O′Malley’s framework.; (1) identifying the initial research questions, (2) identifying relevant studies, (3) study selection, (4) charting the data, and (5) collating, summarising and reporting the results are discussed in reference to this review below.

### 2.1 Identifying the initial research questions

The focus of this review was to identify and compare initiatives that have aimed to improve the process for setting up and conducting clinical trials. These were projects that had been initiated in any jurisdiction, which aimed to make changes across the system rather than improve individual clinical trial management. The primary research question guiding the search was what efforts have been made to improve the clinical trial system efficiency and increase the rate of clinical trials. A secondary research question was developed; what are the success factors of an initiative to increase trial initiation efficiency?

### 2.2 Identifying relevant studies

Key concepts and search terms were developed to capture data that related to clinical trial initiatives across all jurisdictions. Techniques such as Boolean operators were used in conjunction with search terms to focus searches and eliminate irrelevant data. Alternative spellings, ‘program’ and ‘programme’, and alternative terms, ‘clinical study’, ‘clinical trial’, ‘clinical investigation’, were used to account for differences in terminology globally.

Three search strings were used to capture the full scope of the topic, including alternative spellings, and search string 3 was repeated with each continent to reduce location bias;

Search 1: (“initiative” OR “programme” OR “program”) AND (increase “clinical trial” rates OR improve “clinical trial” efficiency OR increase “clinical investigation” rates OR improve “clinical investigation” efficiency OR increase “clinical study” rates OR improve “clinical study” efficiency).

Search 2: (improving “application process” for “clinical investigations”).

Search 3: (increase “clinical trial” rates OR improve “clinical trial” efficiency OR increase “clinical investigation” rates OR improve “clinical investigation” efficiency OR increase “clinical study” rates OR improve “clinical study” efficiency) AND Jurisdiction Name.

To be included in this review, sources needed to be a program or initiative that was implemented to improve the efficiency of the system for conducting clinical trials or to increase the clinical trial rate in a given jurisdiction. This included pharmaceutical and medical device trials in any jurisdiction and for any phase of trial. Initiatives addressing the overall initiation process or a singular aspect were included but were limited to those that reported in English. Sources that focused solely on improving the conduct of individual trials or specific aspects of study design (such as recruitment strategies or protocol amendments) were excluded, as this review aimed to examine broader, system-level initiatives rather than improving individual trial conduct. Similarly, publications that identified barriers or proposed recommendations without describing any implemented changes were excluded, reflecting the review’s focus on practical, applied efforts to improve clinical trial efficiency. There were no limits on the source type or date of the sources, however initiatives identified in the databases and web searches were then added to the list for targeted searches to clarify if there had been any further improvements or iterations.

Bibliographic database searches were conducted in PubMed and ScienceDirect. To identify relevant grey literature, supplementary web searches were performed using GoogleScholar and DuckDuckGo. These searches aimed to capture government reports, regulatory documents, industry publications and other non-indexed sources describing implemented initiatives. PubMed was selected as it indexes the biomedical literature most relevant to clinical trials, while ScienceDirect provides broad coverage of health, regulatory, and management research. As this review aimed to identify implemented initiatives rather than evaluate clinical interventions, extensive grey literature searching was also undertaken to capture reports that may not be in bibliographic databases. Government publications, regulatory documents, reports from recognised professional organisations and industry bodies were considered eligible. Opinion pieces and blogs were used only to provide contextual information for initiatives identified through more authoritative sources and were not considered primary evidence. All the results in the database searches and the first 10 pages of each web search were screened as relevance decreased substantially thereafter and this approach has been recommended in previous evidence synthesis methodology for grey literature searching (8). The searches were supplemented by snowballing of reference lists and Elicit, an AI systematic review tool (9). Elicit has been identified as a useful tool alongside traditional database searches to find additional records and was not used for any further stages (10).

Targeted web searches were used for organisations, programs, and initiatives that were identified during the previous searches. These targeted web searches were used to determine if there were any gaps in knowledge, if there were any more recent updates, or if there was a topic cited in a source that did not provide enough detail. The searches were done with the general search terms and with specific terms of jurisdiction to reduce location bias. The search terms and results for each are shown in the appendix, Additional file 1. DuckDuckGo was chosen for one of the web searches as it does not use any previously collected data and so would reduce personal and location bias further. The searches were first conducted in May 2025 and then repeated to identify new results and perform another round of updates in February 2026.

Consistent with the Joanna Briggs Institute methodology for scoping reviews and PRISMA-ScR guidance, no formal methodological quality assessment of included sources was undertaken because the objective was to map the available evidence rather than evaluate intervention effectiveness (4).

### 2.3 Study selection

Sources were screened using the title/abstract and then added to Covidence (*n* = 120), which removed duplicates (*n* = 26), for full paper screening (11). Due to the large volume of results, especially from web searches, initial title and abstract screening was completed prior to importing records into Covidence. To reduce bias, full-text screening was conducted independently by a second reviewer, with disagreements resolved through discussion.

This process and the number of results at each stage is shown below, adjusted from traditional PRISMA to show the duplicates being removed after title/abstract screening and to show the final ‘bunching’ stage. Data sources that were discussing the same program or initiative were ‘bunched’ to give more detail on the topic and then were further elaborated on with follow up searches. These bunches (*n* = 19) referred to either a once off initiative to make changes to a system or an ongoing program from the sources included (*n* = 27). These were also added to the targeted search list to identify any updates not captured in the search.

### 2.4 Charting the data

The data extracted from each source was a mixture of characteristics (e.g., setting, jurisdiction, and initiative type), and effects of the initiative (e.g., key success factors, implementation challenges, and reported outcomes). The extraction criteria and table were developed prior to extraction. The data was extracted and cross referenced across the sources to include any updates or different perspectives. A follow up search was then conducted on each ‘bunch’ to include any updates. For most bunches there was no update as they had been a once off project or they continued after publication of the source but there had been no further relevant changes. This data was self-reported in the sources or in the follow up searches, except for the key success factors or implementation challenges which were not always stated explicitly as they were not always the primary reported outcome but were often secondary outcomes or described indirectly. The majority of extracted data was qualitative data, with some quantitative data where appropriate and reported. The full extraction table is in the appendix, Additional file 1.

### 2.5 Collating, summarising, and reporting the results

A summary table, Table 1, was created from the extraction table with the main points of each group, focusing on the initiative type and the success factors. The key success factors and implementation challenge from each group were compared to find any which were common. Some of these were specific to the stakeholder involved or jurisdiction it was conducted in and so could not be applied to all future initiatives but is still included. This review did not include analysis of the initiatives’ impact or if trial rates changed as a result. However, some sources did have self-reported outcomes that were included in the extraction table and results.

**Table 1:** Summary table of results.

Initiative/group	Jurisdiction	Initiative type	Aspect addressed	Key success factors
CTTI (20, 28–30)	US	Creates networks, provides guidance and resources, makes recommendations for sponsors	Overall process - has resources for each stage	Multiple stakeholders from diverse backgrounds
ECRIN (12, 31)	EU	Increased engagement, recommendations, policy changes, tools and supports, networking and creation of targeted groups. Contributes to scientific lit	Overall process	International diverse stakeholders, being able to address on an EU level makes a big difference for policy changes and similar and more resources to pool together
EHR4CR (4, 32)	EU	Online tool	Recruitment	Access to resources across EU - hospital records and staff, regulator info
NODES (26)	US	Recruitment centres	Recruitment	Workgroups were able to solve specific problems and develop tools across networks rather than on a trial-by-trial basis, were more efficient, and allowed staff to aid in multiple studies. Guidance was provided to all involved that provided direct support and passed on useful experience
EU Pilot for CI/PS (13, 33, 34)	EU	Engagement with regulators, centralised single submission	Submission	EU level provides ability to make a big change to the regulatory process, willing participants and resources
Trial Innovation Network (21)	US	Network building, contract agreements, design approaches, recruitment support and methods etc.	Overall	Being able to pool resources and expertise and then send sponsors to the right place and then everything learned as the process continued could be shared to everyone
iCARD (22)	US	Network, EHRs system and sharing of data, design support	Dataset merging and designing studies	Pooling of resources and expertise - able to note what challenges were with previous trials and address them, datasets that alone are not significant were combined
Committee on Strategies for Responsible Sharing of Clinical Trial Data (23)	US	Data sharing and guidance on how to do it best	Data sharing	Pooling of resources and expertise from diverse group
Clinical Trials Centre Cologne (CTCC) (14)	Germany	Engagement between sponsors and clinicians, centralised management	Feasibility request and connection between sponsors and CTUs	Being able to create a centralised system and pool resources to facilitate communications made the process consistent and was easier to connect relevant stakeholders
National Clinical Trials Network, Japan (24)	Japan	Centralised review board, standardised systems	Overall process	Stakeholders agree on changes needed, resource pooling
Korea National Enterprise for Clinical Trials (KoNECT) (15)	Korea	Engagement with regulators to develop a standard CT authorisation process, improve infrastructure and policy development	Overall process	Government support and funding, learning from other systems
PRIME (35)	EU	Accelerated pathway	Overall process and increased to market approval	Government backing allowed for big regulatory changes
FDA Total Product Lifecycle Advisory Program (16)	US	Pathway and engagement with regulators	Overall process	Ability to make policy changes and offer increased feedback and priority treatment
Transforming the Activation of Clinical Trials (TACT) (25)	US	Process made more efficient (max happen in parallel, elimination of redundant work), and creation of templates and other work to make it standardised	Trial activation	Ability to make changes across multiple sites (e.g., mutual recognition of radiation safety, review of legal contracts) reduces redundant work and multisite studies do not add unnecessary time. Having a trained PM was the biggest impact
Operational Efficiency Working Group (OEWG) (17)	Korea	Engagement between sponsors and regulators, change in process timeline	Trial activation	Ability to change protocol to allow for pre submission checks (confirm the documents were correct etc.) and make the meetings more frequent to allow for quicker turnaround
Clinical Trials Ontario (27)	Canada	Web-based portal application system	Review from ethics	Centralised system, especially important for multisite trials, use of new technology
Operational Efficiency Working Group, US (18)	US	Increased engagement and redesign of process, dedicated trial staff	Overall process	Pooled resources and unilateral decision-making capabilities, applying lessons learned elsewhere
TrialNation (19)	Denmark	Facilitates engagement across trial, offers guidance, templates, and supports, improves infrastructure	Trial initialisation	Government support, history to build on rather than overhaul
Conditional Early Approval, Japan (36)	Japan	Expedited pathway	Expedited review and conditional approval	Unilateral decision making

The initiative types were extracted from the sources and upon synthesis the category labels were developed. Each initiative included one or more of the eleven initiative types identified, discussed further in the Results section.

## 3 Results

**Figure 1:**
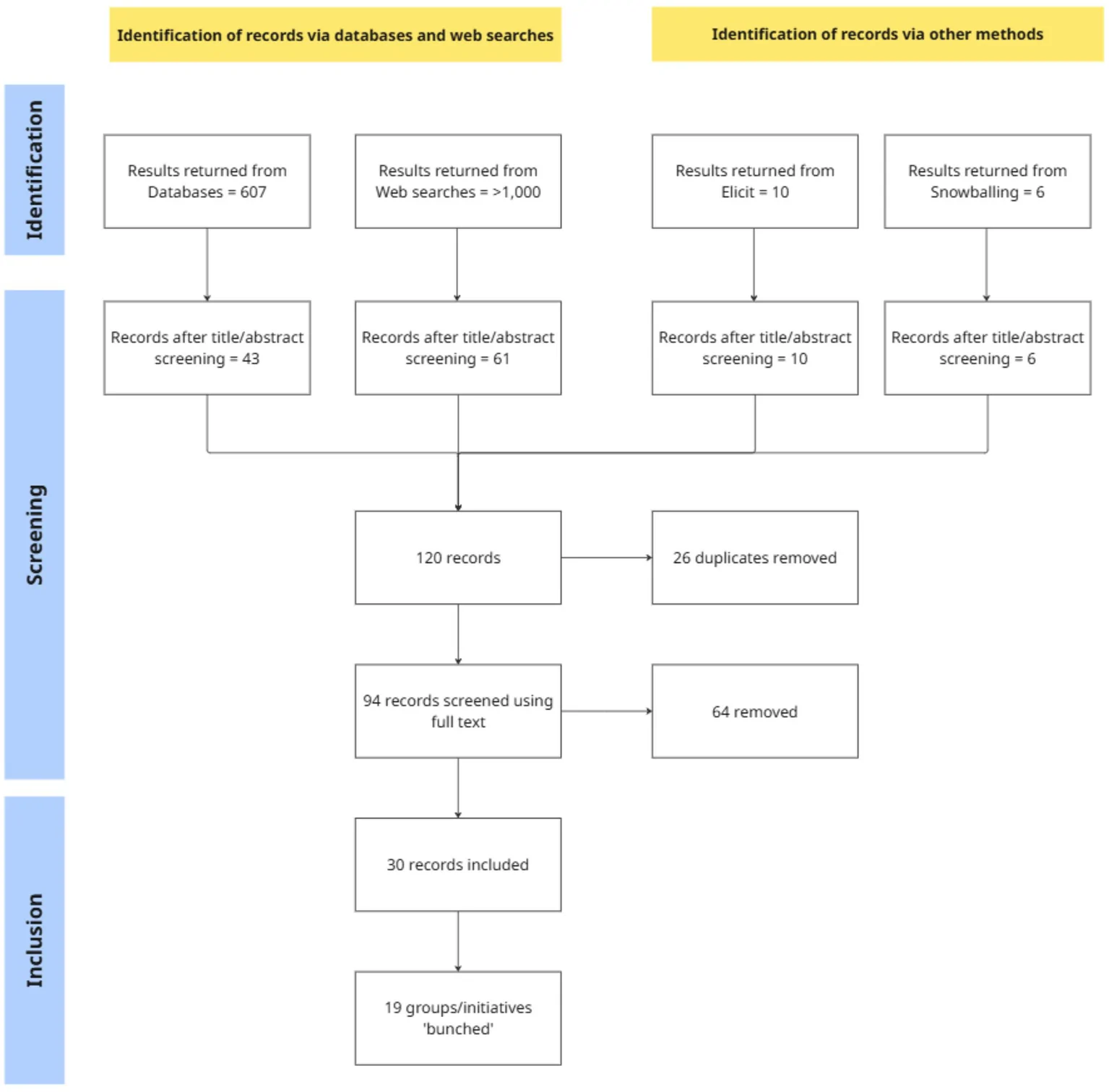
Adjusted PRISMA showing search process.

Screening results are detailed in the PRISMA diagram in Figure 1, which has been adapted to reflect the removal of duplicates after title and abstract screening, as well as the final ‘bunching’ of sources referring to the same initiative. Many records were excluded during the initial screening phase, as they focused on individual trials or general trial guidance rather than system-level initiatives. Given the multiple searches conducted across four databases and search engines, a high number of duplicates were identified and removed. While duplication was expected, repeated searches were necessary to ensure comprehensive coverage, particularly of sources outside the EU and US.

“Clinical trial” is in the title of many published trials and “efficiency” is often used when discussing pharmaceuticals which in part accounts for the large number of results returned that were removed in the title/abstract screening phase. There were also many sources identifying barriers or challenges in running clinical trials or ideas for improving systems, but these were not included.

**Figure 2:**
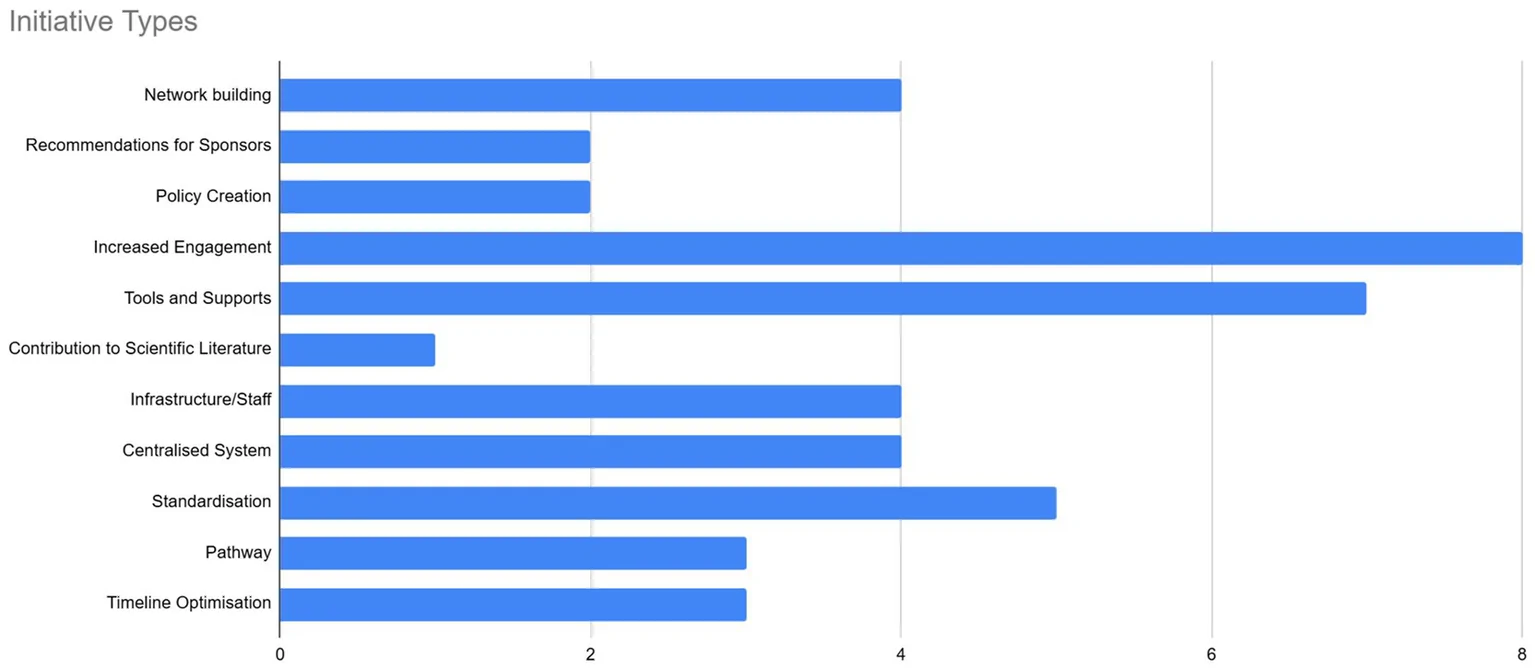
Number of initiatives in each initiative type category.

Across the 19 initiatives, several common characteristics emerged. Most were implemented in high-income countries, were led by regulatory agencies or academic partnerships, and focused on streamlining regulatory approvals and stakeholder collaboration. Few initiatives approached the improvement with a single approach (*n* = 5). While implemented in different ways, the initiatives were categorised to compare and find common approaches. After extracting the initiative type, eleven categories were identified, into which each aspect of improvement fell. These categories can be seen in Figure 2 below. Most initiatives (*n* = 14) included multiple categories, for example both initiatives including recommendations for sponsors also implemented the creation of tools and supports.

The most common category utilised across the 19 initiatives was increased engagement (*n* = 8) (12–19). This was done in different ways, but all involved allowing more engagement between regulators, structured or informal, written or in person, and at different stages. Increased engagement was combined with another approach every time it was implemented as increased engagement alone was determined to not be sufficient for meaningful process improvement. The next most common approach was creation of tools and supports (*n* = 7) (4, 12, 19–23) and standardisation (*n* = 5) (15, 19, 21, 24, 25). The tools and supports created were most often guidance, or development of an online portal. These portals were often paired with standardisation of the process, especially useful for multisite trials to create consistency and reduce redundant work. The standardisation often included creation of templates or checklists for use by sponsors for ease of use and to speed up timelines, especially contract templates. The initiative type for each initiative is shown in a comparison table in the appendix, Additional file 1.

**Figure 3:**
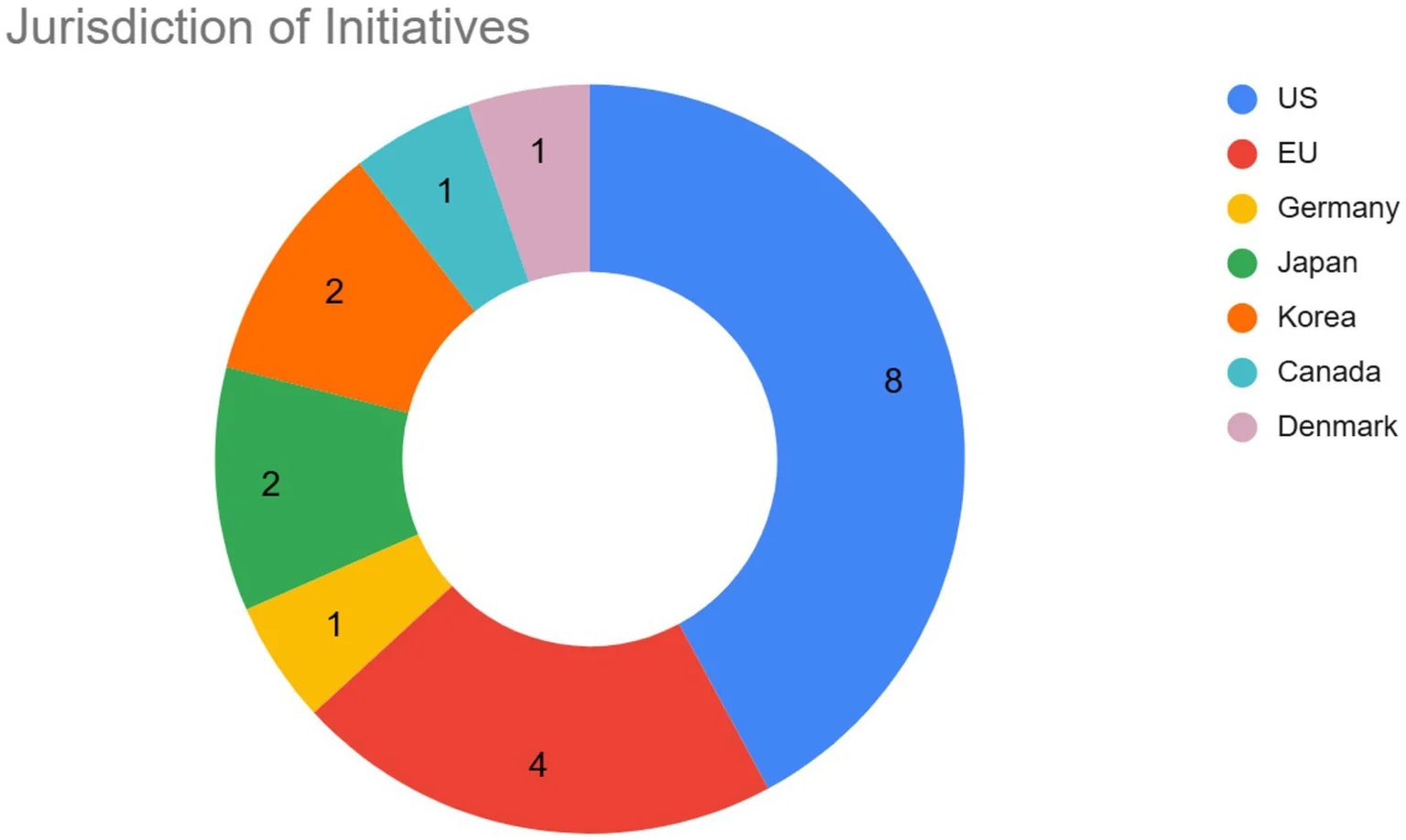
Breakdown of the jurisdictions of the initiatives.

This review brought together initiatives to improve the clinical trials system across jurisdictions around the world. The initiatives varied in scale, settings, and jurisdiction but there were some characteristics that were more common, notably the large number of initiatives conducted in the US (*n* = 8) as shown in Figure 3 (16, 18, 20–23, 25, 26). This is in part due to bias in search strategies and the widespread publishing of American initiatives and the FDA improvements, but it is also due to the work done in the FDA to make changes to the clinical trial system in recent years to compete with other jurisdictions after significant decrease in trial rates.

The choice of approach often varied according to the scale and setting of the initiative, as well as the location and stakeholders involved. In jurisdictions outside the EU, such as the US, Japan, Korea, and Canada, if the stakeholders involved were regulators, they would be able to make changes to how these trials were set up and the process for submission. However, in countries such as Germany, even when regulators are involved, there is a limit to the changes that can be made as they are still a part of the EU regulatory framework.

**Figure 4:**
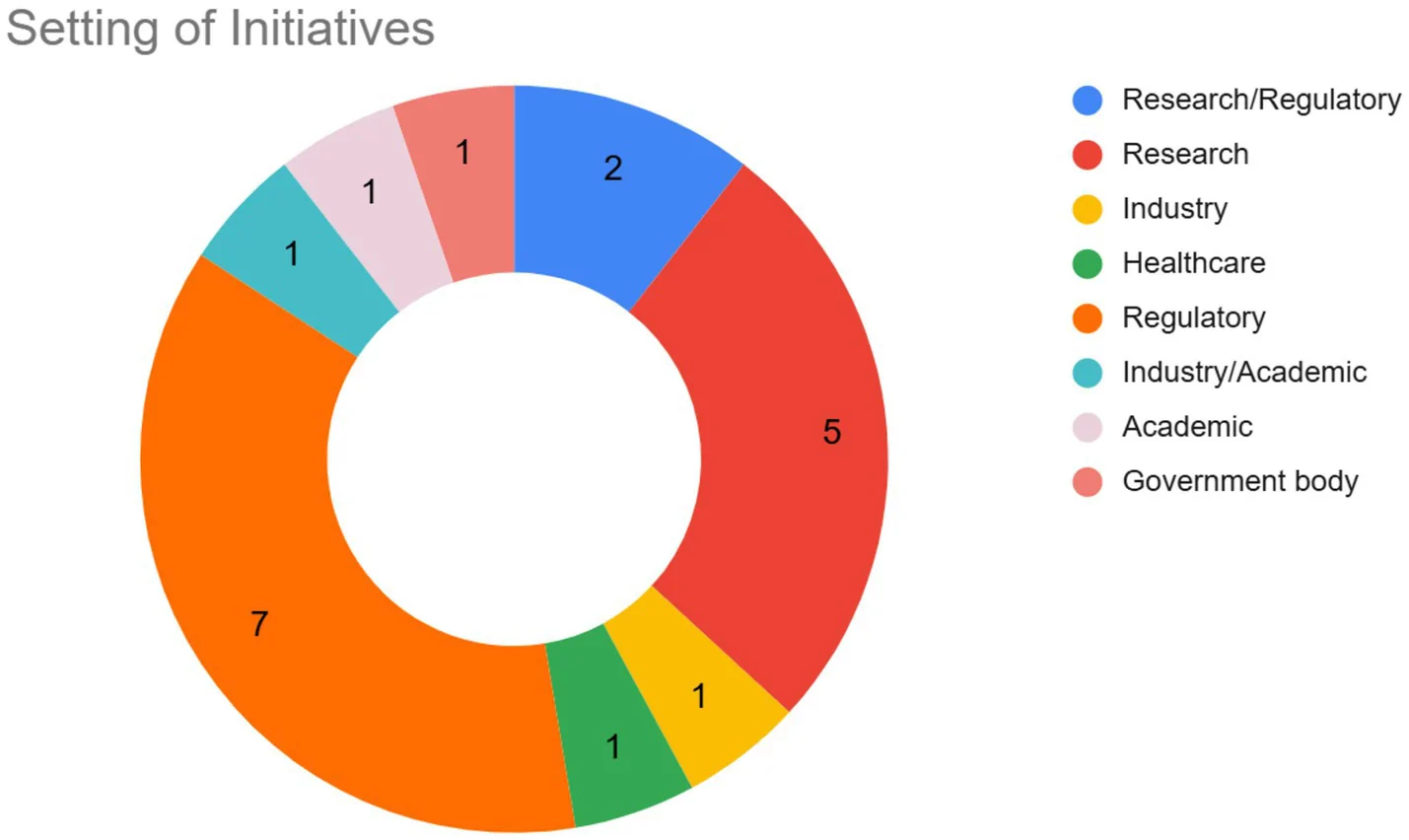
Breakdown of the setting of the initiatives.

Across the initiatives included in this review, the most common setting was regulatory (*n* = 7), shown in Figure 4. This suggests that delays in trial initiation are not due to a lack of effort from regulators but rather reflect broader systemic or structural challenges. The second most common setting was academic or research institutions (*n* = 5), followed by collaborations between research and regulatory bodies. The setting and stakeholder involved influenced the initiative type; if regulators were involved it was possible to increase engagement between them and the sponsor and provided greater control of the process.

The key success factors and implementation challenges were extracted from each initiative. This was sometimes explicitly stated in the source document but was also extrapolated from descriptions. The key success factors and implementation challenges were compared to find which were common across the groups to identify which should be considered for future initiatives, summarised in Textbox 1, 2 Some of the success factors were stakeholder or jurisdiction specific. For example, making direct changes to the submission process requires the involvement of regulatory stakeholders who are both engaged in and supportive of the initiative. The scale of the project and the regulatory framework within a given jurisdiction also influence what changes are possible. In Germany, for instance, one initiative was limited in its ability to implement regulatory changes due to EU-level constraints, and therefore focused its efforts at the site and national levels instead (14). These success factors should be prioritised, and should be used as a framework for future initiatives.

Textbox 1: Key success factors for initiatives to improve clinical trial system efficiency.

**Table:** 

*Key Success Factors:*
Diverse stakeholders that can contribute different perspectives and expertise
Buy-in from all stakeholders across the system
Stakeholders who can influence the system
Use of tools to standardise process and reduce timelines, e.g., templates for timelines
Use of new technology, especially for multisite trials to allow for centralised approach
Resource pooling to allow for more efficient work
Dedicated trial staff that can be given specific training, have protected time, and can share expertise
Learnings taken from previous work applied and new learnings passed on
Government support and funding

Textbox 2: Implementation challenges for initiatives to improve clinical trial system efficiency.

**Table:** 

*Implementation Challenges:*
Usual trial challenges - recruitment, attrition, high costs etc.
Need for consistent research staff
Necessary expertise not always available
Lack of larger network
Regulatory confusion
Infrastructure lacking
Staff do not always have the right training or enough time
Limited influence on the system
Hesitation from others to change the system, taking time to get them onboard and take ownership of the problem

Every group referenced the stakeholders as being a key factor, contributing to the success or limitations of the work. To have a successful project, the stakeholders had to be diverse in order to share expertise and perspectives. The stakeholders needed to have bought into the project and noted in the implementation challenges is when there is hesitation to adopt changes or to accept that changes are needed. The stakeholders also need to be from across the system and have influence over the system being changed. This ‘system’ can be at an institutional level or national level, but the crucial point is to have stakeholders involved who can make the changes planned to ensure that it does not result in recommendations that aren’t adopted or that the changes are only made in a small part or individual basis.

Tools, supports, and guidance were commonly used to standardise and centralise processes, often through new technologies or the development of templates. These strategies help reduce redundant work, which is especially valuable in multisite trials where duplication is more likely (27). Resource pooling also played a key role in improving efficiency, allowing initiatives to combine time, funding, staff, infrastructure, and expertise to drive change more effectively. Sharing expertise across groups was particularly important for learning from past efforts and avoiding repeated mistakes. One of the most effective ways to ensure that lessons are consistently applied is by having dedicated and consistent trial staff. These individuals need proper training and protected time to carry out their work efficiently and to continue learning and improving the process over time.

Government support and funding were particularly valuable in some initiatives, especially those at the national level. Such backing helped ensure that proposed changes were accepted and provided the necessary resources to implement them, an important factor given that these efforts often require significant time and financial investment at the outset.

The implementation challenges identified often mirrored the absence of success factors observed in other projects. For example, limited access to a broader network posed a significant barrier in a smaller initiative (22), while the ability to pool resources across a wider network was cited as a key success factor in larger projects (4, 21, 23).

The US accounted for almost half of the initiatives and groups identified (*n* = 8). This does not take into account individual ‘FDA programs/pathways’ (16) which include multiple initiatives, such as the Breakthrough Device Program, Safer Technologies Program, Total Lifecycle Advisory Program. This shows the deliberate effort coming from the US to improve trial rates. Some of the initiatives included FDA stakeholders but others were on a site level or nationally from an academic standpoint. It is difficult to connect the increase in clinical trials to any single initiative or to prove that the increase would not have happened without the initiative. However, from 2013 to 2023, 20% of high-risk devices approved first in the US were expedited reviews, along with an increase in US-first devices (20). It cannot be said that every one of these sponsors chose the US due to the programs and pathways available, but it is likely that at least a proportion of the increase in trials is due to the introduction of these expedited reviews. Following the introduction of KoNECT, there was an increase in the trial rate and Seoul became the number one city on clinicaltrials.gov, which would have to be attributed to the work of the group at least in part. Other groups had reported outcomes of reduction in approval time or trial initiation which would encourage future trials and increase efficiency of trials that are conducted there.

The number of US based initiatives alone is not enough to determine where the most improvements are being made as this review has some limitations in terms of jurisdictions, with location bias and limited to English-language sources. Quantitative evaluation of initiative effectiveness was inconsistent across the included literature, with many reports describing implementation activities rather than measuring outcomes using comparable metrics. Consequently, direct comparison of effectiveness between initiatives was not possible.

## 4 Discussion

The regulatory process for initiating and conducting clinical trials is lengthy and imposes a significant administrative burden on all parties involved. The combination of high costs and prolonged timelines delays patient access to medical products and discourages developers and researchers who struggle to navigate complex and often costly regulatory requirements. Numerous initiatives, spanning all parts of the clinical trial ecosystem, have aimed to improve the process. Previous reviews have consistently identified regulatory complexity and fragmented governance as barriers to efficient clinical trial initiation. The present review extends these findings by demonstrating that implemented initiatives have moved beyond barrier identification towards practical reforms centred on stakeholder collaboration, process standardisation and shared resources. This provides actionable success factors for policymakers and regulators including the creation of multistakeholder groups to make more effective use of the current resources, standardise and centralise processes, and creation of more opportunities for engagement. A central finding of this review is that the evidence base has moved beyond identifying barriers to clinical trial efficiency. Rather, the remaining challenge lies in implementing and sustaining effective system-level reforms. Although numerous publications describe inefficiencies and recommend improvements, relatively few document implemented initiatives or evaluate their outcomes.

This review identified 27 sources describing 19 distinct initiatives aimed at improving clinical trial system efficiency. Despite their diversity in scope and setting, several common success factors were observed. Chief among them was the involvement of stakeholders who were not only influential within their systems but also actively committed to the change process. Initiatives that engaged stakeholders across sectors, such as regulatory, academic, and clinical, benefited from a broader range of expertise and perspectives, contributing to more practical and widely accepted outcomes. The prominence of stakeholder engagement across initiatives suggests that improvements in trial initiation depend less on isolated procedural changes than on coordinated action across regulatory authorities, sponsors, healthcare organisations and researchers. Because responsibility for trial initiation is distributed across multiple organisations, successful reform requires consensus-building and shared ownership rather than unilateral process redesign.

Resource pooling was another key factor, particularly the sharing of specialised knowledge, trained personnel, infrastructure, and institutional experience. Many initiatives also emphasised the importance of reducing redundant work through process standardisation and centralisation, especially in multisite trials where duplication is common and efficiency gains are essential. The efficient use of resources is incredibly important in an area where high costs have been identified as a barrier, especially for smaller companies or smaller jurisdictions who have limited resources or infrastructure.

The development of new medical devices and pharmaceuticals is inherently complex. Regulatory frameworks and trial initiation processes should facilitate, rather than obstruct, innovation. The findings of this review suggest that many success factors are transferable to future initiatives, starting with assembling the right team. However, the composition of that team, and the strategies employed, must be adapted to the specific regulatory environment and system-level barriers present in each context.

This review had some limitations arising from location and previous searches creating a bias in the returns on web searches, specifically that the results were US centric. This is partially due to the language restriction in this review and from the widespread publishing and discussion of FDA led work. This bias was reduced by including geographical specific searches in the subsequent web searches. The geographical specific searches brought up journals that had not been returned in previous searches as well as studies in that location however it did not completely remove the bias and the prevalence of US publication of work.

The returns were also more inclined towards the FDA and EU Commission publications as they publish widely and are discussed more broadly than some of the other smaller jurisdictions or groups. Owing to the large number of search results it is possible that there are further sources that were missed in this search that were overshadowed by the larger and more widely published initiatives. A major strength of this review is its inclusion of peer-reviewed and grey literature, allowing identification of initiatives that may not have been captured through bibliographic databases alone.

Some groups, such as TrialNation in Denmark, appeared less frequently in the literature, likely because they operate within already well-functioning systems and therefore do not undertake large-scale reform initiatives. Instead, they implement continuous, incremental improvements, building on existing infrastructure without the need for major overhauls. It is possible that other similar groups were not identified in the search, as they may not have published significant updates or large-scale changes. Additionally, while this review included a range of international initiatives, it was not feasible to present detailed accounts of each one, and some nuances from larger or more complex programs may not be fully captured. While this review did not quantitatively assess impact on trial initiation rates, it identifies consistent structural features associated with successful implementation.

The large number of records discussing the desire to improve the clinical trial system, identification of barriers, and recommendations to change them shows there is a willingness to change but there were startlingly few results discussing actual implementation. There was a large disparity between the literature describing barriers and recommending reforms, and the relatively small number of publications describing implemented initiatives. Improving clinical trial efficiency is not limited by knowledge, but by implementation capacity and translating proposed solutions into sustainable changes. The findings indicate that future progress depends on coordinated, system-level adoption of these principles.

## Data Availability

The original contributions presented in the study are included in the article/Supplementary material, further inquiries can be directed to the corresponding author.
